# Frequency-Shifted Interferometry — A Versatile Fiber-Optic Sensing Technique

**DOI:** 10.3390/s140610977

**Published:** 2014-06-20

**Authors:** Fei Ye, Yiwei Zhang, Bing Qi, Li Qian

**Affiliations:** 1 The Edward S. Rogers Sr. Department of Electrical and Computer Engineering, University of Toronto, 10 King's College Road, Toronto, Ontario M5S 3G4, Canada; E-Mails: fei.ye@mail.utoronto.ca (F.Y.); yiwei.zhang@mail.utoronto.ca (Y.Z.); 2 Quantum Information Science Group, Oak Ridge National Laboratory, 1 Bethel Valley Road, Oak Ridge, TN 37830, USA; E-Mail: qib1@ornl.gov

**Keywords:** fiber optics, interferometry, Sagnac interferometer, fiber-optic sensors, sensor multiplexing, cavity ring-down, polarization, Jones calculus

## Abstract

Fiber-optic sensing is a field that is developing at a fast pace. Novel fiber-optic sensor designs and sensing principles constantly open doors for new opportunities. In this paper, we review a fiber-optic sensing technique developed in our research group called frequency-shifted interferometry (FSI). This technique uses a continuous-wave light source, an optical frequency shifter, and a slow detector. We discuss the operation principles of several FSI implementations and show their applications in fiber length and dispersion measurement, locating weak reflections along a fiber link, fiber-optic sensor multiplexing, and high-sensitivity cavity ring-down measurement. Detailed analysis of FSI system parameters is also presented.

## 1. Introduction

As an offshoot of the fiber-optic communications industry, fiber-optic sensing has attracted considerable attention over the years [1–4]. Fiber-optic sensors have many inherent advantages compared to electrical sensors. Made of dielectric materials, fiber-optic sensors are immune to electromagnetic interference (EMI). They can be employed in electric power distribution systems where traditional electrical sensors require laborious insulation due to the high voltage involved [5]. As optical fibers are non-conducting, stable, and chemically inert, they are durable in many harsh environments (e.g., corrosive, high-temperature). Furthermore, fiber-optic sensors can be spark-free, enabling them to be used in monitoring applications for the oil and mining industry where fire hazards are catastrophic. Optical fiber's low loss also allows us to place a sensor head kilometers away from the analyzer without amplifications, which is particularly beneficial for *in situ* measurement over a large area. Fiber-optic sensors are usually more compact, because optical fibers are flexible and lightweight [6,7]. An important feature of fiber-optic sensors is that they have the multiplexing capability—a single system can be used to interrogate multiple sensors linked by off-the-shelf fiber-optic components [1–4,8–14]. To separate signals from different sensors in the sensor network, sensor multiplexing techniques are needed [8–14]. High sensitivity is another advantage of fiber-optic sensors, especially when a sensor is combined with special measurement methods such as the cavity ring-down (CRD) technique [15–29]. Last but not least, driven by the large telecommunication market, the cost of the components used for fiber-optic sensing is decreasing [4]. We have many reasons to believe that the fiber-optic sensing industry shall have a bright future.

Generally speaking, fiber-optic sensing research can be subsumed under two broad categories. One area of research is the design of new sensor types or structures for specific applications. The other area is the development of sensing systems that are intended to effectively detect sensor signals, analyze and interpret them. Frequency-shifted interferometry (FSI) is a fiber-optic sensing technique developed in our group [30–37]. It belongs to the latter category. The setup of FSI is simple—its key components only include a continuous-wave (CW) light source, an optical frequency shifter, and a slow detector. It has been shown to be valuable in many sensing applications such as fiber length and dispersion measurement [30], locating weak reflections along a single fiber link [31,37], fiber-optic sensor multiplexing [32–34], and cavity ring-down measurement [35,36]. FSI can solve some of the issues raised by conventional fiber-optic sensing schemes.

Fiber-optic sensor multiplexing is an important application of FSI. Conventional fiber-optic sensor multiplexing techniques include spatial-division multiplexing (SDM) [10], time-division multiplexing (TDM) [8,9,11], wavelength-division multiplexing (WDM) [8,9], and frequency-division multiplexing (FDM) [13,14]. In SDM, sensing light from a single source is shared by various sensors, and the signal from each sensor is detected by a designated detector. TDM measures the sensor signals by launching optical pulses into an array of sensors with different time delays. The signals from different sensors can be distinguished from their arrival times. In WDM, each sensor must operate within a distinct wavelength window so that sensor signals can be separated from their wavelengths. The frequency-modulated continuous-wave (FMCW) technique, a popular FDM scheme, applies radio frequency (RF) modulation on the source and mixes the signals reflected by the sensors with a local reference. The sensors' signals are differentiated from the beat frequencies. FSI is able to locate sensors from their spatial locations and measure their reflection spectra. This overcomes many limitations faced by conventional fiber-optic sensor multiplexing schemes. Unlike SDM, in which each sensor requires its own detection unit [10], FSI uses one shared detector. An FSI system uses only a low-cost CW light source and a slow detector, instead of the pulsed sources and fast detectors required by TDM. Furthermore, FSI allows the sensors to overlap spectrally. This is not possible for WDM, and FSI can potentially accommodate a larger number of sensors with loosened sensor requirements. Compared to the FMCW technique [13,14], in addition to the avoidance of fast detection electronics, an FSI system does not need any reference signal, and its sensing range is not limited by the coherence length of the light source [14].

FSI provides an alternative way of carrying out cavity ring-down (CRD) measurement [35,36]. In conventional CRD experiments [15–29], an optical cavity is excited by an optical pulse, and the decay rate of the pulse is measured by fast detection electronics. One is able to deduce the loss information of some sample in the cavity from the pulse 1/*e* decay time *τ*, or the so-called CRD time. CRD techniques are frequently used for the spectroscopic analysis of gaseous samples in free-space optical cavities [15–17]. Now there are many demonstrations of fiber-based CRD systems [16,18–27,29]. An advantage of fiber-based CRD techniques is that various kinds of fiber-optic sensors can be incorporated into the system so it may perform very different tasks. Besides spectroscopic analysis, such CRD systems can be used to measure pressure, temperature, strain, refractive index and so on [27]. To perform CRD measurement, FSI does not rely on any optical pulses or fast electronics, since FSI is able to determine the traveled distance as well as the intensity of CW light in a fiber cavity. A CW source and a slow detector may potentially reduce the cost of the system.

In this paper, we provide a review on the development of the FSI technique. We show various ways of implementing FSI and discuss their applications. We also analyze the parameters that affect the performance of FSI systems.

## 2. Principle of Frequency-Shifted Interferometry and Its Implementations

In frequency-shifted interferometry (FSI), a continuous lightwave at frequency *ν*_0_ and a frequency-shifted copy of it at *ν*_0_ + *f*, where *f* is the frequency change, are launched into a common optical path. After the two lightwaves exit the optical path, the frequency of the original lightwave is also shifted to *ν*_0_ + *f* so that it interferes with its frequency-shifted copy, leading to an interference signal *I* that is a sinusoidal function of *f*. By sweeping *f* and recording *I*, one can deduce the optical path length and the interfering light intensity from the sinusoid oscillation frequency *F* and amplitude, respectively. If we send lightwaves (original lightwave and its frequency-shifted copy) through multiple optical paths of different lengths and allow them to interfere in the same fashion as mentioned before (by shifting the original lightwave at *ν*_0_ to *ν*_0_ + *f* after it exits an optical path), the interference signal *I* becomes a summation of sinusoids with distinct oscillation frequencies *F_i_*, with the *i*th sinusoidal component produced by lightwaves passing through the *i*th optical path. A Fourier transform on *I* can separate the interference signal contributed by light from each path, and in the Fourier spectrum, we can learn the length and optical loss associated with an individual path from the location and amplitude of a Fourier component, respectively. Above idea proves to be very useful for fiber-optic sensing [30–37]. In this section, we shall explain the operation of several FSI implementations and show their applications.

### 2.1. Frequency-Shifted Sagnac Interferometer

**Figure 1.**
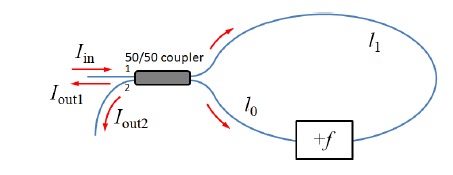
A frequency-shifted Sagnac interferometer formed by connecting the output of a 50/50 fiber directional coupler with an optical frequency shifter asymmetrically.

The earliest FSI developed is an asymmetric frequency-shifted Sagnac interferometer [30,38–40] as shown in Figure 1. The interferometer is constructed by connecting two output ports of a 50/50 fiber directional coupler with an optical frequency shifter (e.g., an acousto-optic modulator, or AOM). The asymmetry arises due to unequal fiber section lengths between the frequency shifter and the fiber coupler. Suppose an input electric field *E_in_*=*E*_0_ exp(2*πνt* + *ϕ*_0_) is launched into the interferometer from port 1, where *E*_0_ is the field amplitude, *ν* is the optical frequency, and *ϕ*_0_ is some initial phase. The two counter-propagating fields at port 1 are:

(1.a) $$E_{cw1}={\left(\sqrt{\gamma }\right)}^{2}E_{0}\cdot \exp\left\{i\cdot \left[2\pi \left(\nu +f\right)\;t+\frac{2\pi \;nl_{1}\nu }{c}+\frac{2\pi \;nl_{0}(\nu +f)}{c}+\phi_{0}+\frac{\pi }{2}\right]\right\}$$

(1.b) $$E_{ccw1}={\left(\sqrt{\gamma }\right)}^{2}E_{0}\cdot \exp\left\{i\cdot \left[2\pi \left(\nu +f\right)\;t+\frac{2\pi \;nl_{0}\nu }{c}+\frac{2\pi \;nl_{1}(\nu +f)}{c}+\phi_{0}+\frac{\pi }{2}\right]\right\}$$

and the fields at port 2 are:

(2.a) $$E_{cw2}={\left(\sqrt{\gamma }\right)}^{2}E_{0}\cdot \exp\left\{i\cdot \left[2\pi \left(\nu +f\right)\;t+\frac{2\pi \;nl_{1}\nu }{c}+\frac{2\pi \;nl_{0}(\nu +f)}{c}+\phi_{0}\right]\right\}$$

(2.b) $$E_{ccw2}={\left(\sqrt{\gamma }\right)}^{2}E_{0}\cdot \exp\left\{i\cdot \left[2\pi \left(\nu +f\right)\;t+\frac{2\pi \;nl_{0}\nu }{c}+\frac{2\pi \;nl_{1}(\nu +f)}{c}+\phi_{0}+\pi \right]\right\}$$

where *γ* = 0.5 is the fiber coupler split ratio, *f* is the frequency shift induced by the frequency shifter, *n* is the effective refractive index of the fiber mode, *c* is the speed of light in vacuum, and *l_i_* and *l*_1_ are the fiber section lengths as shown in the figure. Here ${\left(\sqrt{\gamma }\right)}^{2}$ is used in above equations to emphasize that each field component passes the fiber coupler twice before the interference. The term *π*/2 in (1) and *π* in (2.b) account for the phase shift acquired when the light crosses the coupler ports. It can be shown that the differential intensity between port 1 and port 2 is a sinusoidal function of *f* [30,33]:

(3) $$\begin{array}{ll} \Delta I=I_{\text{out}1}-I_{\text{out}2} & \propto 2\gamma^{2}E_{0}^{2}\cos\left[2\pi \frac{n\;\left(l_{1}-l_{0}\right)}{c}f\right]\\ & \propto 2\gamma^{2}E_{0}^{2}\cos\left[2\pi \cdot F\cdot f\right]\\ & \propto 2\gamma^{2}E_{0}^{2}\cos\Delta \phi \end{array}$$

where *F* = *n*(*l*_1_ − *l*_0_)/*c*, and Δ*ϕ* = 2*π·F·f* is the phase difference between the two interfering fields. The amplitude of Δ*I* is proportional to the interfering light intensity. Although *F* has the unit of time, if one considers Δ*I* as a function of *f*, *F* becomes the oscillation frequency of the sinusoid and it is proportional to *n*(*l*_1_ − *l*_0_). This relation can be used to measure fiber length and dispersion [30].

**Figure 2.**
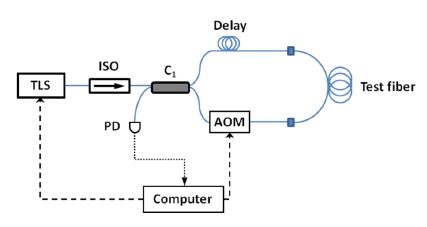
Experimental setup of a frequency-shifted Sagnac interferometer for fiber length and dispersion measurement [30]. TLS: CW tunable laser source; ISO: fiber-optic isolator; PD: photodetector; C_1_: 50/50 fiber direction coupler; AOM: acousto-optic modulator.

In [30], a CW laser is used as the light source, and an acousto-optic modulator (AOM) is used as the optical frequency shifter as shown in Figure 2. A single photodetector is used to record the sinusoidal interference signal from one output port of the interferometer as the frequency shift *f* is swept between 50 and 56 MHz. The interference signal is thus a sinusoidal with a DC offset, from whose period fiber length can be deduced. Spools of single-mode fibers with lengths ranging from 5 m to 60 km were tested. The measurement results were in good agreement with those obtained from tape measurement or an Agilent 86037C chromatic dispersion test system. As group delay can be determined by scanning the input wavelength and recording the optical lengths, chromatic dispersion of the test fiber can also be calculated [30]. The setup of a frequency-shifted Sagnac interferometer is simpler than common fiber optical length measurement techniques such as the optical time-domain reflectometry (OTDR) [41], optical coherence-domain reflectometry (OCDR) [42,43], or optical frequency-domain reflectometry (OFDR) [44,45]. The wide measurement range demonstrated by FSI is superior to that of OCDR (∼10 cm) [42,43] and OFDR (∼km) [44,45].

### 2.2. Linear Frequency-Shifted Sagnac Interferometer with Multiple Reflections

**Figure 3.**
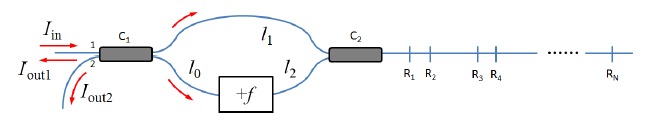
A linear frequency-shifted Sagnac interferometer with multiple reflectors. C_1_ and C_2_: 50/50 fiber directional couplers, R*_i_*: the *i*th reflector.

Alternatively, an FSI can adopt a configuration akin to a Mach-Zehnder (MZ) interferometer as shown in Figure 3, where C_1_ and C_2_ are 50/50 fiber couplers, and R*_i_* are reflectors (*i* = 1,2,3,…*N*). Despite its MZ-like appearance, we would refer to this configuration as the linear frequency-shifted Sagnac interferometer, because its operation principle is still based on that of a frequency-shifted Sagnac interferometer.

Suppose CW input light at *ν* is launched into the interferometer from port 1 of C_1_. It is divided equally into two parts, and the two lightwaves continue to propagate towards the reflectors. Each reflector R*_i_* shall introduce four reflected components at frequencies *ν*, *ν* + *f*, *ν* + *f*, and *ν* + 2*f*. The frequencies of the reflected components are determined by the number of times light goes through the frequency shifter. Note the two reflected components at *ν*+ *f* are contributed by the lightwaves that pass the frequency shifter exactly once. They are equivalent to the two counter-propagating lightwaves in a frequency-shifted Sagnac interferometer discussed in the previous section. Therefore, a reflector R*_i_* in Figure 3 essentially offers us a frequency-shifted Sagnac interferometer in which the two fiber sections connecting the frequency shifter have lengths *l*_0_ and *l*_1_ + 2*L_i_* + *l*_2_, where *l_i_* are the fiber lengths labeled in the figure, and *L_i_* is the fiber length between C_2_ and the reflector. The differential interference signal from all the reflectors is a summation of sinusoids [33]:

(4) $$\begin{array}{ll} \Delta I & \propto \overset{N}{\underset{i=1}{\text{∑}}}A_{i}\cdot \cos\left[2\pi \cdot \frac{n\;\left(l_{1}+2L_{i}+l_{2}-l_{0}\right)}{c}\cdot f\right]\\ & \;\propto \overset{N}{\underset{i=1}{\text{∑}}}A_{i}\cdot \cos\left[2\pi \cdot \frac{2n\;L_{i}}{c}\cdot f\right]=\overset{N}{\underset{i=1}{\text{∑}}}A_{i}\cdot \cos\left(2\pi \cdot F_{i}\cdot f\right) \end{array}$$

where *A_i_* is some amplitude proportional to the reflectivity of the *i*th reflector R*_i_*, and *F_i_* = 2*nL_i_*/*c*. In the second line of equation, we make the assumption 2*L_i_* ≫ *l*_1_ + *l*_2_ − *l*_0_ so that (*l*_1_ + 2*L_i_* + *l*_2_ − *l*_0_) ≈ 2*L_i_*. As *L_i_* is unique for each reflector, *F_i_* are also different from one another. If one records Δ*I* as a function of *f*, and process the data with Fourier transform, the sinusoidal components can be separated in the frequency domain *F*. Frequency *F* can be easily converted into distance by applying *L* = *cF*/ 2*n*. Therefore, we can essentially separate the signals from different reflectors in the spatial domain. The amplitude of the peak at frequency *F_i_* is proportional to the interfering light intensity from the *i*th reflector. By recording the Fourier peak amplitudes at various *F_i_*'s over a range of input wavelengths, we are able to construct the reflection spectra of all the reflectors.

The remaining reflected components at *ν* and *ν* + 2*f* produce DC signals at C_1_ output ports, which are effectively eliminated by the differential measurement. The intermixing of the fields at *ν*, *ν* + *f*, and *ν* + 2*f* generates beat frequencies at *f* and 2*f*, but they average out to zero when a slow detector is used to measure Δ*I* (e.g., if *f* ∼ 100 MHz, and the detector has a bandwidth of 10 MHz).

**Figure 4.**
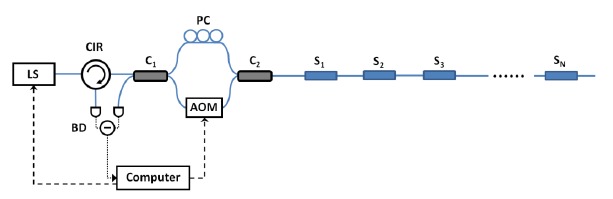
Typical setup of a linear frequency-shifted Sagnac interferometer for fiber-optic sensor multiplexing. LS: light source; CIR: circulator; BD: balanced detector; AOM: acousto-optic modulator; PC: polarization controller; C_1_ and C_2_: 50/50 fiber directional couplers; S*_i_*: *i*th sensor (of reflection type).

A linear frequency-shifted Sagnac interferometer is a useful tool for multiplexing reflection-type fiber-optic sensors, since it can separate the sensor signals from the spatial domain and measure sensors' reflection spectra. As mentioned in the Introduction, FSI offers many advantages over conventional fiber-optic sensor multiplexing schemes. Figure 4 shows a typical linear frequency-shifted Sagnac interferometer. The sensing light from a light source (LS) is launched into the interferometer through a fiber-optic circulator (CIR). An AOM is used as a frequency shifter. The differential interference signal is measured by a balanced detector (BD). A polarization controller (PC) may be added to adjust the interference fringe visibility. This system can be applied to many applications depending on the types of sensors employed.

Reflection-type sensors such as the ones based on fiber Bragg gratings (FBGs) are particularly suitable for FSI measurement [32,34]. In [32], an array of 10 FBG sensors was interrogated by a linear frequency-shifted Sagnac interferometric system. Despite gratings' overlapping Bragg wavelengths, FSI can resolve the reflection spectrum for each and every sensor in the array. A signal-to-noise ratio (SNR) as high as 48 dB was attained. As FBG sensors are strain/temperature sensors, this FSI system may potentially be used in areas such as civil structural health monitoring or fire prevention [9,12]. With specially designed FBG sensors, this type of FSI configuration can be applied to liquid level sensing [34]. The aluminum-coated high attenuation FBGs (HAFBGs) used in [34] may be heated by in-fiber light, and they show significantly larger spectral change when heated in the air compared to that in liquid. FSI can measure the reflection spectra of such sensors in an array to unambiguously determine whether a sensor is immersed in liquid.

Transmission-type sensors can also be interrogated by FSI systems if each sensor is used in conjunction with a reflector. For example, when the sensor array consists of gas cells with partial reflective mirrors, FSI is capable of identifying and quantifying chemical gases at different locations. In [33], an array of 3 gas cells was successfully measured by FSI. Spectral lines of carbon monoxide and acetylene at different concentrations were clearly identified from the measured gas cell absorption spectra. A minimum detectable acetylene concentration of 230 ppm with a 3-cm gas cell was achieved [33]. It is comparable to the detection limit of a TDM system with wavelength modulation (150 ppm with a 2.5-cm gas cell) [46].

**Figure 5.**
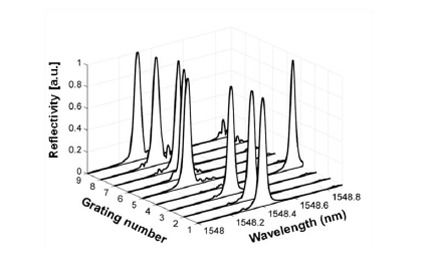
Overlapping FBG spectra measured by an FSI system that uses a light source consisting of a broadband ASE source and a tunable filter.

In most FSI fiber-optic sensor multiplexing demonstrations [32–34], a tunable laser was used. However, it is not mandatory. An incoherent amplified spontaneous emission (ASE) source paired with a tunable filter may be incorporated into an FSI system as the light source. Figure 5 shows the spectra of an array of FBGs measured by an FSI system using such a light source. The full width at half maximum of the filter is 0.05 nm. As we can see, FSI can still provide individual grating spectra to high accuracy although the sensor Bragg wavelengths are similar to one another. As a tunable filter can be tuned at a high scan frequency (∼ kHz), using an ASE source and a tunable filter may increase the measurement speed and reduce the system cost. However, it sacrifices the SNR and spectral resolution, since a tunable laser offers higher spectral power and a narrow line width.

### 2.3. Single-Arm Frequency-Shifted Interferometer with Multiple Reflections

**Figure 6.**
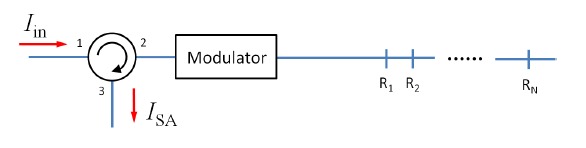
A single-arm frequency-shifted interferometer. R*_i_* is the *i*th reflector.

Based on sideband interference, an FSI system with only one interferometer arm can also be built [37]. This single-arm FSI (SA-FSI) configuration (see Figure 6) is simple and more compact compared with a linear frequency-shifted Sagnac interferometer. Let the input field be *E*_0_ *e*^*i*2*πνt*^. The modulator (an intensity modulator or phase modulator) in SA-FSI introduces sideband signals. When a phase modulator is employed, the output field of the modulator can be expressed as:

(5) $$\begin{array}{ll} E(t) & =E_{0}\cdot \exp\left\{i\left[2\pi vt+M\;\sin\left(2\pi \;ft\right)\right]\right\}\\ & =E_{0}\cdot \exp(i\;2\pi v\;t)\overset{\infty }{\underset{m=-\infty }{\text{∑}}}J_{m}(M)\exp(i\;2\pi \;\text{mf t}) \end{array}$$

where *M* is the modulation index, and *J_m_*(*M*) is the Bessel function of the first kind. With properly chosen modulation parameters, higher order sidebands can be suppressed. Now let us focus on the interference of the first order sideband at *ν* + *f* at port 3 of the circulator. The central band light at *ν* passes the modulator twice (on one occasion before it enters and reflector array, and on another occasion when it returns to the modulator after reflection at R*_i_*) and produces two components at *ν* + *f* for R*_i_*. These two components are equivalent to the two interfering lightwaves at *ν* + *f* in a linear frequency-shifted Sagnac interferometer. The output intensity of the SA-FSI system *I_SA_* is:

(6) $$I_{SA}\propto I_{DC}+\overset{N}{\underset{i=1}{\text{∑}}}R_{i}\cos\;\left(2\pi \cdot \frac{2nL_{i}}{c}\cdot f\right)$$

where the DC component *I_DC_* is the result of the central band signal, and *L_i_* is the distance between *R_i_* and the modulator. Again, a Fourier transform can separate the interference signals from different reflectors. A bandpass filter can be used to select the interference signal at *v* + *f*.

In [37], a 10-GHz LiNbO_3_ phase modulator (PM) was used as the modulator, and a few weak FBGs were introduced as reflectors in the single fiber link. As a PM can be driven at a much higher RF frequency than a AOM, we were able to sweep the frequency shift *f* between 4.5 and 5.5 GHz, in steps of 1 MHz. This corresponds to a frequency sweep range Δ*f* of 1 GHz, which is much larger than that of a typical AOM (20 MHz). As shall be explained in part 3 of this paper, spatial resolution is inversely proportional to Δ*f*. A spatial resolution of 0.3 m can be achieved with this SA-FSI, and this is a significant improvement compared to the resolution of an FSI system using an AOM (∼5 m).

### 2.4. FSI-Based Cavity Ring-Down Technique

The principle of FSI can be applied to CRD measurement [35,36]. Instead of monitoring the decay rate of an optical pulse in a cavity, FSI-based CRD (FSI-CRD) technique measures the decay rate of CW light in the cavity without the need of any fast electronics.

**Figure 7.**
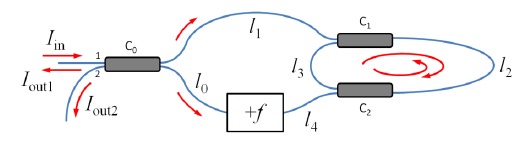
An FSI-CRD system with a fiber loop cavity. C_0_: 50/50 fiber directional coupler; C_1_ and C_2_: highly unbalanced fiber directional couplers.

Figure 7 shows the basic setup of an FSI-CRD system with a fiber loop cavity. It is essentially a frequency-shifted Sagnac interferometer that incorporates a fiber loop ring-down cavity (RDC). The RDC is constructed by two highly unbalanced fiber directional couplers C_1_ and C_2_ (e.g., 99.5/0.5 couplers). When CW light is sent into the interferometer from port 1 of C_0_, lightwaves start to circulate in the RDC in opposite directions. A small fraction of the light exits the RDC each time when the light completes a round trip. If the cavity length *d* = *l*_2_ + *l*_3_ is longer than the coherence length of the light source, interference takes place at C_0_ between counter-propagating lightwaves that exit the RDC after the same number of round trips. As the differential interference signal contains a sinusoidal component for each round trip number *m* (*m* = 0,1,2,3,…), Δ*I* is [35]:

(7) $$\begin{array}{ll} \Delta I & \propto \overset{\infty }{\underset{m=0}{\text{∑}}}I_{m}\cdot \cos\left\{2\pi \frac{n[l_{1}+l_{2}+m(l_{2}+l_{3})+l_{4}-l_{0}]}{c}f\right\}\\ & \propto \overset{\infty }{\underset{m=0}{\text{∑}}}I_{m}\cdot \cos\left\{2\pi \frac{n[Ls+md]}{c}f\right\}\\ & \propto \overset{\infty }{\underset{m=0}{\text{∑}}}I_{m}\cdot \cos\left\{2\pi \cdot F_{m}\cdot f\right\} \end{array}$$

where the length constant *L_S_* = *l*_1_+ *l*_2_+ *l*_4_− *l*_0_, *I_m_* is the amplitude of the sinusoid component, and *F_m_* = *n*(*L_s_* + *md*)/*c*. Clearly, *F_m_* increases by *nd*/*c* as *m* increases. Due to RDC loss, *I_m_* undergoes an exponential decay. Let us assume that there is some sensing element in fiber section *l*_2_ with attenuation coefficient *α* and an interaction length *l*. It can be shown that *I_m_* can be expressed as [35]:

(8) $$I_{m}=I^{\prime }\cdot \exp\left(-\frac{-\ln\kappa_{c}+\alpha l}{d}\cdot L_{t}\right)$$

where *I*′ is some initial amplitude, *κ_c_* is the transmittance of an empty RDC (an RDC without any sensing element), and *L_t_* = *md* is the distance traveled by light in the RDC. Similar to conventional CRD measurement in which an exponential decay is obtained, the Fourier transform of Δ*I* also shows a series of exponentially decaying peaks, but in our case, the peaks decay as a function of distance instead of as a function of time. Analogous to the CRD time, we may define a CRD distance for FSI-CRD as:

(9) $$\Lambda =\frac{d}{-\ln\kappa_{c}+\alpha l}$$

By finding Λ, we can deduce the loss introduced by the sensing element in the RDC.

**Figure 8.**
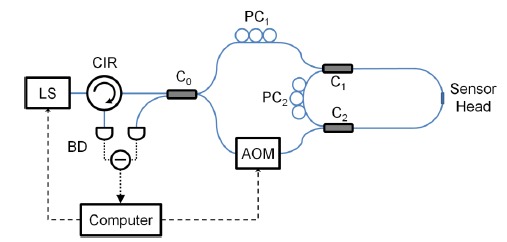
A typical FSI-CRD sensing system [35,36]. LS: light source; CIR: circulator; BD: balanced detector; AOM: acousto-optic modulator; C_0_: 50/50 fiber directional coupler; C_1_ and C_2_: highly unbalanced fiber directional couplers.

Figure 8 shows the setup for a typical FSI-CRD sensing experiment. The light source (LS) could be either a tunable laser [35] or a broadband source [36]. The frequency shifter employed is again an AOM. We have demonstrated that this system can accurately measure fiber bend loss introduced in the RDC [35]. To compare the performance of FSI-CRD with that of conventional ones, we conducted FSI-CRD evanescent-field sensing experiments [36].

Some reviews on fiber-based CRD techniques can be found in references [27,29]. Two research groups have used conventional CRD techniques to measure the absorption of 1-octyne [23,25]. In [23], optical pulses were injected into a 2.2-km RDC formed by a pair of 99/1 fiber directional couplers. A fiber taper with a diameter of 10 μm was used in the RDC as the sensing element. The cavity loss change was induced by the evanescent-field absorption caused by 1-octyne at the fiber taper section. An estimated minimum detecTable 1-octyne concentration of 1.05% was reported. In another demonstration [25], instead of a taper, a long-period grating (LPG) pair was employed in a similar but shorter RDC (∼28 m), where the evanescent field absorption occurred between the two LPGs. A range of 1-octyne concentrations (dissolved in decane) between 0 and 40% was tested, and a minimum detectable concentration of 0.62% was reported [25]. To evaluate the performance of our technique, we measured the absorption of 1-octyne solutions between 0% to 5% (1-octyne dissolved in decane) with our FSI-CRD system. The CW sensing light was produced by a low-coherent C-band amplified spontaneous emission (ASE) source. A 5-cm-long fiber taper (10 μm in diameter) was spliced into a fiber loop cavity formed by two 99.5/0.5 fiber directional couplers. The cavity loss was measured as a function of 1-octyne concentration. It was found that a minimum detectable 1-octyne concentration of 0.29% could be achieved. To the best of our knowledge, this is the lowest 1-octyne detection limit in literature for fiber-based CRD systems [23,25]. Compared with the result in [23], the improvement in the detection limit could be due to a longer light-sample interaction length (provided by a longer taper) and a shorter but more stable RDC (∼47 m). Although the interaction length (∼16 cm) in [25] is longer, our taper may produce a larger evanescent field compared with that offered by the fiber cladding modes excited by the LPGs in [25]. As our RDC is less lossy, it may also contribute to the superior performance. We used the same FSI-CRD setup for refractive index sensing [36]. A minimum detectable refractive index change of 1 × 10^−4^ was achieved for sodium chloride solutions, which is comparable to the result (3.2 × 10^−5^) attained by a conventional fiber-based CRD system using a much longer taper [26]. Note that in our experiments, the data sampling rate was only 100 kS/s, whereas in conventional CRD experiments, fast detection electronics (>20 MS/s) are needed to capture the change of optical pulses with durations of ∼10 ns [15].

## 3. FSI System Parameters

### 3.1. Spatial Resolution and Spatial Sensing Range

In an FSI system, the spatial resolution is the minimum resolvable separation between two reflectors (minimum resolvable optical path length difference). In fiber-optic sensor multiplexing, this parameter dictates how closely sensors (such as FBGs) can be distributed along a fiber. In a linear frequency-shifted Sagnac interferometer or a SA-FSI system, the spatial resolution *δL* is proportional to the resolution of sinusoidal components' oscillation frequency *F* after the FT: *δL* = *c*/(2*n*)· *δF*. By the theory of discrete Fourier transform, *δF* = 1/Δ*f*, where Δ*f* is the frequency sweep range of the frequency shifter, so that [33]:

(10) $$\delta L=\frac{c}{2n\Delta f}$$

As fiber is flexible and can be wound, *δL* is not necessarily the minimum sensor physical separation.

The spatial sensing range is the maximum distance between the furthest sensor and the system at which reliable measurement can be made (maximum measurable optical path length). Since *F_i_* is proportional to *L_i_* by Equation (4), the further the reflector, the higher the frequency *F_i_*. Nyquist theorem suggests that we need to sample Δ*I_i_* at a sampling rate at least twice as high as *F_i_*. That is, the maximum *F* we can measure *F_max_* = 1/(2*f_step_*), where *f_step_* is the frequency shifter sweep step. The spatial sensing range is thus [33]:

(11) $$L_{\max}=\frac{c}{{4\text{nf}}_{\text{step}}}$$

In practice, system loss must also be considered when one estimates the maximum sensing distance, if certain signal-to-noise ratio (SNR) is required.

As we have seen, both spatial resolution and spatial sensing range are determined by the sweep parameters of the optical frequency shifter. The following relation holds:

(12) $$\frac{L_{\max}}{\delta L}\propto \frac{\Delta f}{f_{\text{step}}}$$

Depending on the requirements of a specific application, the spatial resolution and spatial sensing range can be optimized by choosing appropriate frequency shifter sweep range and sweep step, respectively. For example, if we have Δ*f* = 20 MHz and *f_step_* = 0.04 MHz, the spatial resolution and spatial sensing range are ∼5 m and ∼1293 m, respectively [33].

### 3.2. Dispersion Effects

Up to now, the analysis of FSI has been under the assumption of monochromatic input light. To see the effects of dispersion in FSI, let us suppose that the input light is broadband and that it has a uniform spectrum centered at *λ*_0_ with a bandwidth of Δ*λ*. The differential interference signal Δ*I* then becomes an integral over all the wavelength components. In the case of a linear frequency-shifted Sagnac interferometer, the differential interference signal Δ*I_i_* contributed by a single reflector R*_i_* can be expressed as:

(13) $$\begin{array}{ll} \Delta I_{i} & \propto \int_{\lambda_{0}-\Delta \lambda /2}^{\lambda_{0}+\Delta \lambda /2}\;I_{i}\cos\left[2\pi \frac{2n\;(\lambda )L_{i}}{c}f\right]d\lambda \\ & \propto I_{i}\int_{\lambda_{0}-\Delta \lambda /2}^{\lambda_{0}+\Delta \lambda /2}\;\cos\left[2\pi \frac{2n_{0}L_{i}+D2L_{i}\cdot (\lambda -\lambda_{0})\cdot c}{c}f\right]d\lambda \\ & \propto \Delta \lambda I_{i}\cos\left(2\pi \frac{2n_{0}L_{i}}{c}f\right)\cdot \text{sinc}\left(2\pi \cdot \;DL_{i}\Delta \lambda \;\cdot f\right) \end{array}$$

where *I_i_* is the interference signal intensity, and the optical path length (OPL) for a spectral component at *λ* has been written as 2*n*(*λ*)*L_i_* + *D*2*L_i_* (*λ* − *λ*_0_) *c*, and *D* is the group velocity dispersion parameter. It can be seen that the integral is essentially a summation over a continuous range of frequency components *F*(*λ*) = 2*n*(*λ*)*L_i_*/*c*. After Fourier transform, these frequency components form a broadened peak around the central wavelength component *F*(*λ*_0_). Also note that the cosine term *I_i_* cos[2*π*(2*n*_0_*L_i_*/*c*)*f*] in Equation (13) is the usual interference signal contributed by the component at *λ*_0_, and the dispersion effect due to bandwidth Δ*λ* is included in the sinc function. The oscillation of this sinc function is much slower than the cosine term. The Fourier transform of Δ*I_i_* is the convolution between the Fourier transforms of a cosine and a sinc function.

**Figure 9.**
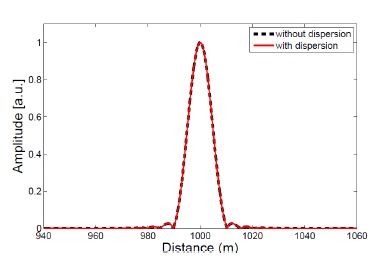
Comparison between the normalized Fourier peaks with and without dispersion effects. The thick black dashed curve is the Fourier peak contributed by light at *λ*_0_ without dispersion, while the red curve is the Fourier peak computed from Equation (13). The Fourier transform was calculated with a Hann window and a fast Fourier transform size of 2^20^.

As a numerical example, let us consider the dispersion effect in a linear frequency-shifted Sagnac interferometer built with standard SMF-28 fibers. At *λ*_0_ = 1550 nm, the fiber group velocity dispersion parameter *D* = 16.2 ps/(nm·km). Suppose the source bandwidth Δ*λ* = 35 nm, *L_i_* = 1 km, and the frequency shift is swept from 90 to 110 MHz in steps of 0.04 MHz by an AOM. The Fourier peak computed with the dispersion effect is almost identical to that computed without dispersion, as can be seen in Figure 9. This can be easily understood when we compare the OPL difference between light traveling at *λ*_0_ and at other wavelengths—the OPL difference is on the order of only 0.17 m, which is well below the width of the Fourier peak (∼10 m).

### 3.3. System Crosstalk

Three types of crosstalk may influence the performance of an FSI system, including spectral shadowing effect, discrete Fourier transform resolution, and unwanted reflections among reflectors.

#### 3.3.1. Spectral Shadowing Effect

**Figure 10.**
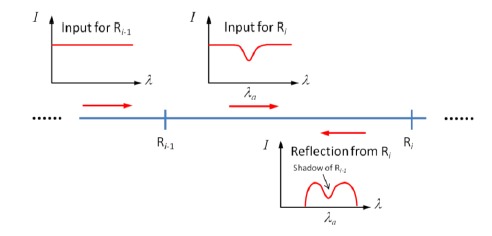
Spectral shadowing effect. The (*i* − 1)th sensor has a spectral feature at *λ_a_*, and it casts a shadow on the *i*th sensor. As a result, the reflection from R*_i_* contains the spectral information of R*_i_*_−1_.

When multiple sensors in a serial array have overlapping spectral features, spectral shadowing effects take place. To reach a specific sensor, sensing light needs to pass all upstream sensors. Therefore, the input light of the *i*th sensor carries the spectral characteristics of previous (*i* − 1) sensors, and is thus “shadowed” by them (see Figure 10).

As a form of crosstalk, spectral shadowing effects are not unique to FSI, but are common in conventional fiber-optic sensing systems [9]. In an FSI sensing system, spectral shadowing effects can be effectively removed [33].

In FSI, the system can resolve the reflection spectrum for every individual sensor. The spectral shadow experienced by the *i*th sensor can be removed by using the spectrum of the (*i* − 1)th sensor. The interference signal amplitude *A_i_* of the *i*th sensor (*i* ≥ 2) can be written as:

(14) $$A_{i}={\left(\prod_{j=1}^{i-1}T_{j}\right)}^{2}\;R_{i}$$

where *T_j_* = 1 – *R_j_* is the transmittance of the *j*th sensor and *R_i_* is the reflectivity of the *i*th sensor. The multiplications in the braces represent the shadow caused by the previous (*i* – 1) sensors. It can be shown that the reflectivity *R_i_* is:

(15) $$\begin{array}{ll} R_{i} & \propto \frac{A_{i}}{A_{i-1}}\cdot \frac{R_{i-1}}{T_{i-1}^{2}}\\ & \propto \frac{A_{i}}{A_{i-1}}\cdot \frac{R_{i-1}}{{\left(1-R_{i-1}\right)}^{2}} \end{array}$$

As there is no spectral shadow for the first sensor, *R*_1_ can be obtained directly from the reflection spectrum measured for the first sensor. The actual reflection spectra of subsequent sensors can be calculated sequentially by using Equation (15).

#### 3.3.2. Discrete Fourier Transform Crosstalk

**Figure 11.**
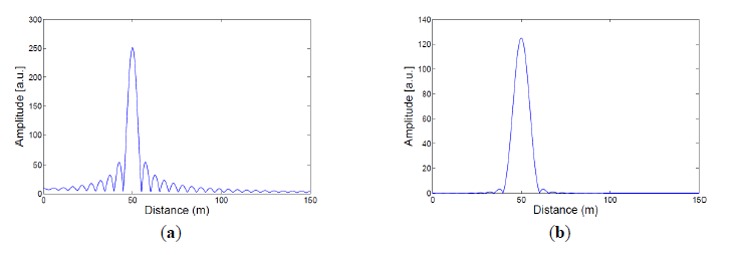
The effects of windowing in DFT. Given the same interference signal Δ*I*, a Hann window can effectively suppress the side lobes of a Fourier peak. (**a**) DFT spectrum of Δ*I* with a rectangular window. (**b**) DFT spectrum of Δ*I* with a Hann window.

The interference signal Δ*I* acquired in an FSI system is a set of finite data. After the data set is processed by discrete Fourier transform (DFT), the Fourier peaks in the Fourier spectrum have finite widths [47,48]. DFT crosstalk occurs when two Fourier peaks become overlapped in the spectrum, that is, when the frequencies of two sinusoidal components are very close to each other. It is undesirable as one Fourier peak may distort the shape of the other, and vice versa. DFT crosstalk can be remedied if the sweep range Δ*f* is increased or an appropriate window function (e.g., a Hann window) is applied in the DFT calculation [47,48]. Figure 11 compares the DFT spectra of a given Δ*I* computed with and without a special window function. As can be seen from Figure 11a, if DFT is calculated directly (*i.e*., with a rectangular window), large side lobes appear about the Fourier peak, and their amplitude decreases slowly as one moves away from the peak center. On the other hand, when a Hann window is employed in DFT (see Figure 11b), the side lobes are effectively suppressed. Less DFT crosstalk is expected if we apply a proper window, such as the Hann window, in the calculation of DFT.

#### 3.3.3. Unwanted Reflections Among Reflection Sites

**Figure 12.**
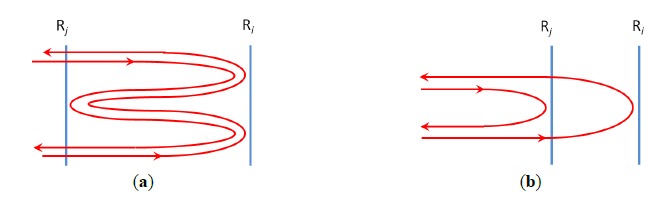
The effects of unwanted reflections. (**a**) Multiple reflections between a pair of sensors. (**b**) Interference between lightwaves reflected by different sensors.

If the sensing system contains multiple sensors in series, multiple reflections or inter-sensor interference may occur, which presents another potential source of crosstalk in FSI systems. Sensing light may make multiple trips between a pair or more sensors. If the two interfering sensing lightwaves follow the same routes and are bounced back and forth between sensors as shown in Figure 12a, secondary peaks may appear in the Fourier spectrum after Δ*I* is processed with DFT. These peaks may overlap and distort the primary Fourier peaks contributed by light interference without multiple reflections. Choosing low-reflectivity sensors may reduce the crosstalk caused by this effect [9].

Light reflected by two different sensors R*_i_* and R*_j_* may also interfere, if the coherence length of the light source is longer than twice the sensor separation (see Figure 12b). The interference signal can be shown to have the form of *A_i_A_j_* cos Δ*ϕ*, where *A_i_* and *A_j_* are the amplitudes of the interfering fields, and Δ*ϕ* is the phase difference between them. Table 1 summarizes the contributions given by such undesirable interference for a linear frequency-shifted Sagnac interferometer system. The left column of the table shows the paths interfering field components follow, while the right column lists the corresponding phase difference between the field components. The interference signals in the first two rows of the table introduce Fourier peaks at *F_j_* and *F_i_* which are indistinguishable from the actual sensor signals. The interference signal in the 3rd row of the table is a DC component, and the 4th row contribution is of low frequency near DC for small separation between R*_j_* and R*_i_*. The crosstalk presented by the first two rows of the table can be minimized by employing a low-coherent light source (e.g., a broadband source) or by increasing sensor separations. The contributions of the DC and slow frequency interference signal from table's last two rows can be minimized by choosing sufficiently long fiber length between the first sensor and the system.

### 3.4. Polarization Effects

Many interferometric fiber-optic sensing systems are susceptible to polarization effects [49–51]. Polarization fading occurs when the interfering fields' polarization states are misaligned. As an interferometric technique, FSI's performance is also influenced by light polarization. We developed a model based on Jones calculus [52–54] to study the polarization effects in FSI systems. In our experiments, the FSI systems are built with single-mode fibers (SMFs), which have very low polarization-dependent loss. Nonlinear effects are also negligible as a result of the low optical power admitted into the system (<10 mW). Therefore, it is reasonable to assume that the Jones matrices of the components in the FSI system are elements of SU(2), the special unitary group [54–57]. That is, we assume there is no fiber nonlinearity, no polarization-dependent loss, and the loss term has been factored out so that the Jones matrices are unitary. We also assume that there are no Faraday effects and that the fiber directional couplers do not alter light polarization. We seek conditions under which the interference fringe visibility can be maximized.

Under our assumptions above, the Jones matrix for an optical component in an FSI system can be generally written as [55]:

(16) $$\begin{array}{ll} U & =\left[\begin{array}{cc} a & b\\ -b* & a* \end{array}\right]\\ & =\left[\begin{array}{cc} e^{i\xi }\cos\eta & e^{i\varsigma }\sin\eta \\ -e^{-i\varsigma }\sin\eta & e^{-i\xi }\cos\eta \end{array}\right] \end{array}$$

where *a* = *e^iξ^* cos*η* and *b* = *e^iζ^* sin*η* are complex numbers (*a*,*b*∈**C**) satisfying |*a*|^2^ +|*b*|^2^ = 1. The matrix can be characterized by *ξ*, *η*, and *ζ*, which are three real continuous parameters (*ξ*, *η*, *ζ* ∈ **R**) [55] The matrix *U* can alternatively be written as:

(17) $$U=\left[\begin{array}{cc} e^{i\rho } & 0\\ 0 & e^{-i\rho } \end{array}\right]\;\left[\begin{array}{cc} \cos\eta & \sin\eta \\ -\sin\eta & \cos\eta \end{array}\right]\;\left[\begin{array}{cc} e^{i\psi } & 0\\ 0 & e^{-i\psi } \end{array}\right]$$

where *ρ* and *ψ* are two real parameters such that *ρ* + *ψ* ≡ *ξ* and *ρ* + *ψ* ≡ *ς*. We can recognize immediately that *U* is in fact constructed by a polarization rotator with a rotation angle *η* sandwiched between two phase shifters with phase shifts 2*ρ* and 2*ψ*, respectively [53].

The Jones matrix of a cascaded system is the product of its constituents' Jones matrices [52,53]. As matrix multiplications are closed for elements of SU(2), the Jones matrix of a cascaded system whose constituents' matrices belong to SU(2) is also an element of SU(2), and it can be characterized by 3 real parameters. Mathematically, the matrix product *U*_1_*U*_2_*U*_3_*U*_4_*U*_5_…*U_N_* = *U* ∈ SU(2), given *U_i_* ∈ SU(2).

A FSI system is bidirectional, since light propagates through the same optical elements from both directions. Given the Jones matrix *U* of an optical element for forward light propagation, we also need to find the corresponding Jones matrix *UŪ* for backward propagation. The entries of *Ū* is related to those of *U* by the relation *Ū_ij_* = (−1)*^i^*^+^*^j^ U_ji_* [58,59]. With *U* as in Equation (16), the backward propagation Jones matrix is therefore:

(18) $$\begin{array}{ll} Ū & =\left[\begin{array}{cc} a & b*\\ -b & a* \end{array}\right]\\ & =\left[\begin{array}{cc} e^{i\xi }\cos\eta & e^{-i\varsigma }\sin\eta \\ -e^{i\varsigma }\sin\eta & e^{-i\xi }\cos\eta \end{array}\right] \end{array}$$

It can also be shown that the backward propagation Jones matrix for a cascaded system has the property [58]:

(19) $$\bar{U_{N}U_{N-1}U_{N-2}\cdots U_{1}}=\bar{U_{1}}\;\bar{U_{2}}\;\bar{U_{3}}\;\cdots \bar{U_{N}}$$

where *U_i_* are the forward Jones matrices of components in the system. With (16)-(19), we can start to model the polarization effects in FSI systems.

#### 3.4.1. Polarization Model for a Frequency-Shifted Sagnac Interferometer

For a frequency-shifted Sagnac interferometer, suppose the Jones matrices for clockwise propagation and counterclockwise propagation are *M_c_* and *M_a_*, respectively. They can be expressed as:

(20a) $$M_{c}=\left[\begin{array}{cc} e^{i\xi }\cos\eta & e^{i\varsigma }\sin\eta \\ -e^{-i\varsigma }\sin\eta & e^{-i\xi }\cos\eta \end{array}\right]$$

(20b) $$M_{a}=\bar{M_{c}}=\left[\begin{array}{cc} e^{i\xi }\cos\eta & e^{-i\varsigma }\sin\eta \\ -e^{i\varsigma }\sin\eta & e^{-i\xi }\cos\eta \end{array}\right]$$

**Figure 13.**
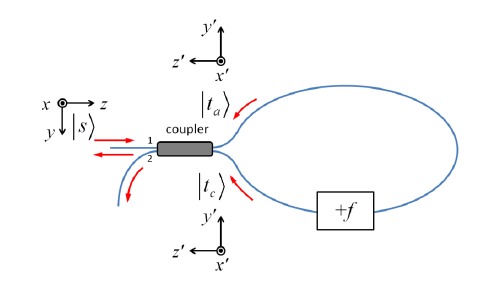
Input and output polarization of a frequency-shifted Sagnac interferometer. The coordinate system for the input polarization state ∣*s*〉 is *x*-*y*-*z*, and the coordinate system for the output states∣*t_c_*〉 and ∣*t_a_*〉 is *x*´-*y*´-*z*´.

For an arbitrary input Jones vector ∣*s*〉 = [*s_x_ s_y_*]^T^, the output polarization of clockwise propagation is ∣*t_c_*〉 = *M_c_*∣*s*〉, and that of counterclockwise propagation is ∣*t_a_*〉 = *M_a_*∣*s*〉. To optimize the interference fringe visibility, we require ∣*t_c_*〉 = ∣*t_a_*〉, where the two polarization states are defined in their corresponding local coordinates *x*´-*y*´-*z*´ as shown in Figure 13. This condition is satisfied for an arbitrary ∣*s*〉 when *M_c_* = *M_a_*, which implies that *e^iζ^* sin*η* = *e*^−^*^iζ^* sin*η*, that is, *ζ* = *kπ*, where *k* is an integer (*k*∈**Z**).

#### 3.4.2. Polarization Model for a Linear Frequency-Shifted Sagnac Interferometer

**Figure 14.**
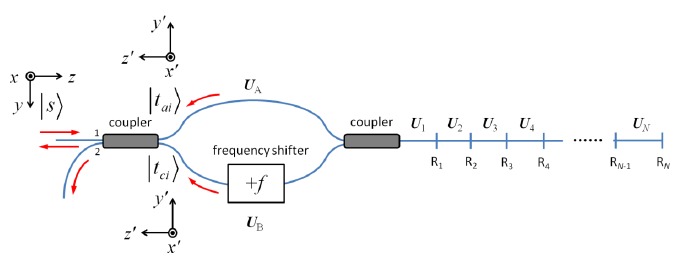
Input and output polarization of a linear frequency-shifted Sagnac interferometer. The coordinate system for the input polarization state ∣*s*〉 is *x*-*y*-*z*, and that for the output polarization states ∣*t_ci_*〉 and ∣*t_ai_*〉 from the *i*th sensor R*_i_* is the local coordinate system *x*´-*y*´-*z*´.

The system for a linear frequency-shifted Sagnac interferometer is more complicated. As shown in Figure 14, the fiber section between any two adjacent sensors may possess a random Jones matrix *U_i_* (assumed to belong to SU(2) in our analysis), and given the same input ∣*s*〉, the output polarization states for different sensors are in general different. We may write the clockwise and counterclockwise Jones matrices for the system, *M_ci_* and *M_ai_*, for the *i*th sensor as:

(21a) $$M_{\text{ci}}=U_{B}U_{\text{arrayi}}U_{A}$$

(21b) $$M_{\text{ai}}=\bar{U_{A}}U_{\text{arrayi}}\bar{U_{B}}$$

where *U_A_* and *U_B_* are the Jones matrices for the fiber sections shown in Figure 14, and $U_{\text{arrayi}}=\left(\bar{U_{1}}\bar{U_{2}}\bar{U_{3}}\cdots \bar{U_{i-1}}\bar{U_{i}}\right)\left(U_{i}U_{i-1}U_{i-2}\cdots U_{2}U_{1}\right)$. But as $M_{\text{ai}}=\bar{M_{\text{ci}}}=\bar{U_{A}}\bar{U_{\text{arrayi}}}\bar{U_{B}}$, by Equation (21b), we can conclude that *U_arrayi_* = *U̅_arrayi_*. This means that the (1,2) entry of *U_arrayi_* must be a real number, namely, [*U_arrayi_*]_12_ ∈ **R**. Without loss of generality, we may assume that:

(22) $$U_{\text{arrayi}}=\left[\begin{array}{cc} e^{i\xi_{0}}\cos\eta_{0} & \sin\eta_{0}\\ -\sin\eta_{0} & e^{-i\xi_{0}}\cos\eta_{0} \end{array}\right]$$

and that:

(23) $$U_{A}=\left[\begin{array}{cc} e^{i\xi_{A}}\cos\eta_{A} & e^{i\varsigma_{A}}\sin\eta_{A}\\ -e^{-i\varsigma_{A}}\sin\eta_{A} & e^{-i\xi_{A}}\cos\eta_{A} \end{array}\right],\;\;U_{B}=\left[\begin{array}{cc} e^{i\xi_{B}}\cos\eta_{B} & e^{i\varsigma_{B}}\sin\eta_{B}\\ -e^{-i\varsigma_{B}}\sin\eta_{B} & e^{-i\xi_{B}}\cos\eta_{B} \end{array}\right]$$

To optimize the interference fringe visibility for an arbitrary input polarization state ∣*s*〉, we require that *M_ci_*∣*s*〉 = ∣*t_ci_*〉 = ∣*t_ai_*〉 *M_ai_*∣*s*〉, where ∣*t_ci_*〉 and ∣*t_ai_*〉 are the output polarization states for clockwise propagation and counterclockwise propagation, respectively. Again, this implies [*M_ci_*]_12_ ∈ **R**, the (1,2) entry of the Jones matrix *M_ci_* is a real number. By substituting Equations (22) and (23) into Equation (21), we may find the expressions for *M_ci_* and *M_ai_*. The system is now characterized by 8 parameters (*ξ*_0_, *η*_0_, *ξ_A_*, *ς_A_*, *η_A_*, *ξ_B_*, *ς_B,_* and *η_B_*).

It was found that if we can control *U_A_* and *U_B_* (*i.e.*, if we can adjust *ξ_A_*, *ς_A_*, *η_A_*, *ξ_B_*, *ς_B,_* and *η_B_*), it is possible to simultaneously maximize the interference fringe visibility for all the sensors, as long as we can satisfy either of the two sets of conditions:

(24) $$\{\begin{array}{c} \cos\eta_{A}=0\\ \cos\eta_{B}=0\\ \sin(\varsigma_{B}+\varsigma_{A})=0 \end{array}\text{or}\{\begin{array}{c} \sin\eta_{A}=0\\ \sin\eta_{B}=0\\ \sin(\xi_{B}-\xi_{A})=0 \end{array}$$

This suggests that in a practical FSI sensor interrogation system, we may keep the setup to the left of coupler C_2_ in the control center, and we can optimize the interference fringe visibility for all the sensors, although we do not have access to the sensor array.

#### 3.4.3. Polarization Model for an FSI-CRD System

**Figure 15.**
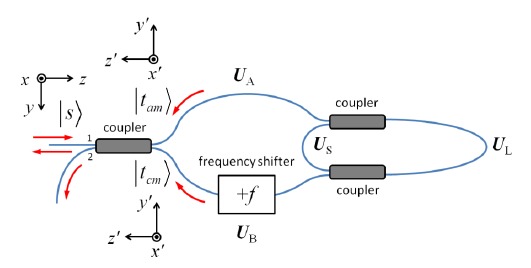
Input and output polarization of an FSI-CRD system. The coordinate system for the input polarization state ∣*s*〉 is *x*-*y*-*z*, and the coordinate system for the output states after *m* round trips ∣*t_cm_*〉 and ∣*t_am_*〉 is *x*´-*y*´-*z*´.

In an FSI-CRD system, the polarization state evolves as light makes multiple passes through the ring-down cavity. We can denote the Jones matrices of the system components as shown in Figure 15. For a given round trip number *m* (*m* = 0,1,2,…), the clockwise propagation Jones matrix *M_cm_* and the counterclockwise propagation Jones matrix *M_am_* can be written as:

(25) $$M_{\text{cm}}=U_{B}{\left(U_{L}U_{S}\right)}^{m}U_{L}U_{A}=U_{B}U_{\text{RDCm}}U_{A}$$

(26) $$\begin{array}{ll} M_{\text{am}} & =\bar{U_{A}}{\left(\bar{U_{L}}\;\bar{U_{S}}\right)}^{m}\bar{U_{L}}\;\bar{U_{B}}\\ & =\bar{M_{\text{cm}}}=\bar{U_{B}U_{\text{RDCm}}U_{A}}\\ & =\bar{U_{A}}\;\bar{U_{\text{RDCm}}}\;\bar{U_{B}} \end{array}$$

where *U_RDCm_* = (*U_L_U_S_*)*^m^U_L_* accounts for the multiple passes of light through the RDC.

We wish to optimize the interference fringe visibility for every *m*. Following similar arguments as in the previous section, we can show that having polarization control over *U_A_* and *U_B_* alone does not guarantee optimal fringe visibility for all the interference signals (for every *m*), unlike the case of a linear frequency-shifted Sagnac interferometer. We also need to control the polarization inside the RDC. The simplest way to achieve fringe visibility optimization is to use two polarization controllers, one for *U_A_* (or *U_B_*) and the other for *U_S_*. As *U_A_*, *U_B_*, *U_L_*, and *U_S_* are assumed to be elements of SU(2) in our model, they are all invertible. One can first adjust *U_A_* (or *U_B_*) so that *U_B_U_L_U_A_* = *I*, where *I* is the identity Jones matrix, and then adjust *U_S_* to make *U_L_U_S_* = *I*. Once this is achieved, we have $U_{\text{RDCm}}=\bar{U_{\text{RDCm}}}=I$ and *M_cm_* = *M_am_* = *I*, so that the output polarization states ∣*t_cm_*〉 and ∣*t_am_*〉 will be identical to the input polarization state ∣*s*〉.

## 4. Conclusions

In summary, fiber-optic sensing has become an important frontier of the sensing industry. Frequency-shifted interferometry is a versatile addition to the tool box. With the help of a CW light source, a frequency shifter, and a slow detector, FSI is able to undertake very different tasks. An FSI system can be used to measure fiber length and dispersion [30], and to locate faults or weak reflections along a fiber link [31,37]. An important application of FSI is in fiber-optic sensor multiplexing [32–34]. Fiber-optic sensors of both transmission- and reflection- types can be employed in the system. FSI can separate sensor signals in the spatial domain, and measure their spectra. The spectra of reflection-type sensors such as FBGs can be measured directly by FSI [32,34]. For transmission-type sensors, the transmission spectra can be obtained by placing a reflector after each sensor [33]. In general, a fiber-optic sensor that produces spectral or transmission/reflection loss change in response to the measurand change can be interrogated by an FSI system. These sensors can operate at similar wavelengths, which relaxes the requirements on the sensors and enables the system to accommodate a larger number of sensors. If a fiber-loop ring-down cavity is embedded into the system, FSI is capable of performing high-sensitivity cavity ring-down analysis [35,36]. FSI-CRD measures the decay rate of CW light in the RDC, and therefore, it does not require any optical pulse or fast electronics. In this paper, we have presented different configurations of FSI, explained the operation principles, and analyzed the system parameters that affect the performance of FSI systems. The information can serve as a guide for the design and optimization of FSI systems targeting various applications. We believe that with the continual expansion of the fiber-optic sensing market, FSI shall make greater contributions to the community.

## Figures and Tables

**Table 1.** Contributions of interference caused by undesirable reflections for a linear frequency-shifted Sagnac interferometer system [33]. With reference to Figure 3, path A is the fiber section between the two couplers that does not have the frequency shifter, whereas path B is the other fiber section that contains the frequency shifter.

**Path of the Interfering Field Components**	**Δ*ϕ***
path A → R*_j_* → path B and path B → R*_i_* → path A	2*πn*(2*L_i_*+*l*_1_+*l*_2_−*l*_0_)*f/c*+2*πn*(2*L_i_*−2*L_j_*)*ν*_0_/*c*
path A → R*_i_* → path B and path B → R*_j_* → path A	2*πn*(2*L_j_*+*l*_1_+*l*_2_−*l*_0_)*f/c*+2*πn*(2*L_j_*−2*L_i_*)*ν*_0_/*c*
path A → R*_j_* → path B and path A → R*_i_* → path B	2*πn*(2*L_i_*−2*L_j_*)*ν*_0_/*c*
path B → R*_j_* → path A and path B → R*_i_* → path A	2*πn*(2*L_i_*−2*L_j_*)*f/c*+2*πn*(2*L_i_*−2*L_j_*)*ν*_0_/*c*
